# Three-dimensional CBCT based evaluation of the inferior part of the maxillary sinus: Retrospective Study

**DOI:** 10.1038/s41598-020-78156-x

**Published:** 2020-12-03

**Authors:** Jeong-Hyun Lee, Won-Jeong Han, Jong-Tae Park

**Affiliations:** 1 Department of Oral Anatomy, Dental College Dankook Institute For Future Science and Emerging Convergence, Dan-Kook University, Cheonan, 330-714 South Korea; 2 Department of Dentomaxillofacial Radiology, Dental College, Dan-Kook University, Cheonan, 330-714 South Korea

**Keywords:** Anatomy, Medical research

## Abstract

The maxillary sinus is the largest of the four paranasal sinuses in humans, and its close proximity to the teeth means that caution is required during dental treatment. In particular, implant surgeries involving the maxillary posterior teeth should include evaluating the inferior part of the maxillary sinus. The purpose of this study is to evaluate the differences by comparing the inferior part of the maxillary sinus based on the nasal cavity floor (NCF) between patients (male 30, female 30) genders through the use of the three-dimensional (3-D) program that can facilitate 3-D visualizations. The present study results obtained from 3-D visualizations using cone beam computed tomography (CBCT) data showed that the inferior part of the maxillary sinus was mostly larger in males than in females. In addition, the utilization of 3-D visualization data was more likely to assure accuracy than when using data obtained by two-dimensional (2-D) imaging. Therefore, 3-D visualizations of the inferior part of the maxillary sinus will contribute to accurate analyses of its anatomical structure during implant surgery and other operations. Further studies utilizing 3-D visualization will yield useful fundamental data and guidelines for future research.

## Introduction

The maxillary sinus is the largest of the four paranasal sinuses in humans, and its close proximity to the teeth means that caution is required during dental treatment^1^. Of them, the inferior part of maxillary sinus is an important anatomical structure considered first in clinical practice. In particular, implant surgeries involving the maxillary posterior teeth should include evaluating the inferior part of the maxillary sinus. Implant surgery involves performing a surgical procedure for a missing tooth^2^. Severe loss of alveolar bone necessitates the use of bone grafting and maxillary sinus floor augmentation procedures for the maxilla^3^. However, maxillary sinus floor augmentation frequently leads to complications such as perforation in the inferior part of the maxillary sinus, excessive bleeding, and hematoma^4,5^. Hence, dental radiographs have been used to analyze the anatomical structure of the inferior part of the maxillary sinus, including to predict the prognosis of implant surgery^3,6–12,14–16^. However, it is difficult to differentiate between the maxillary sinus floor and the Schneiderian membrane on radiographs^7^. Accurate measurements are especially important for surgical treatment, and the use of cone-beam computed tomography (CBCT) for observing the anatomical structure of the inferior part of the maxillary sinus is still in its infancy.

CBCT allows the assessment of complicated anatomical structures that are difficult to observe on panoramic scans due to the presence of overlapping structures. CBCT has previously been used in the treatment of lesions, trauma, and congenital malformation surgery, but it is now also being widely used in dental clinics^3,6–12,14–16^. Studies have used CBCT in maxillary sinus floor augmentation for measuring the height of the residual bone crest^6,8,9^, the thickness of the lateral maxillary sinus bone wall^6,10,11^, and the angle of the palatal-nasal recess (PNR)^3,11^ in the inferior part of the maxillary sinus. Therefore, the inferior part of maxillary sinus is an anatomically and clinically important structure. Nevertheless, studies reported so far measured CBCT in two-dimensional (2-D) without visualizing it in three-dimensional (3-D)^3,6–12,14–16^. CBCT does not suffer from panoramic overlap, analyses might not be accurate without utilizing 3-D visualization^7^. Hence, studies utilizing the 3-D visualization capabilities of CBCT are required.

The nasal cavity floor (NCF) is a baseline that is currently considered important in implant surgery. In particular, NCF acts as a criterion for bone implantation to enable implant placement during maxillary sinus floor augmentation^17^. In addition, it is possible to prevent complications such as bleeding, pain, cross-contamination, implant displacement, and sinusitis when maxillary sinus floor augmentation and implant surgery are performed based on NCF^18^. As such, it is commonly used for implant surgery based on NCF. However, only cross- sectional area of CBCT has been measured in most current studies.

The purpose of this study is to evaluate the differences by comparing the inferior part of the maxillary sinus based on the NCF between patients’ genders through the use of the Mimics program that can facilitate 3-D visualizations. In addition, we aimed to determine the accuracy and difference between the measured length in 2-D of previous studies and the measurement conducted via 3-D visualization in this study. Therefore, this study measured the distance between the zygomatic arch to examine the facial width, with an aim to determine the relationship between facial width and the inferior part of the maxillary sinus.

## Materials and methods

### Study participants

Among the malocclusion (class I, II, III) patients in their twenties who visited the Orthodontics Department of Dankook University Dental Hospital, CBCT data were obtained from 60(male 30, female 30) adults without missing teeth, asymmetry, or systemic disease from the Oral Maxillofacial Radiology Department. In addition, the G-Power 3.1 (HHU, England) program was used to calculate the number of samples for the study participants.

The study was conducted after IRB (Dankook University Dental Hospital, approval no. DUDH IRB 2015-12-022) had been approved. This study is a retrospective analysis of radiological imaging data which were obtained from the completed check-up processes. Thus, an application for waiver of consent was requested and was approved by the institutional review committee of Dankook University Dental Hospital.

### Methods

#### Generation of 3-D images

The CBCT data of the participants were obtained in DICOM format from the images captured by a scanner (Alphard 3030, Asahi, Kyoto, Japan). CBCT scanning was performed with a slice increment of 0.39 mm, slice thickness of 0.39 mm, and a matrix of 512 pixels × 512 pixels. The DICOM images were used to generate 3-D images of the maxillary sinus using the Mimics 3-D imaging program (version 22.0, Materialise).

#### Parameters

**Figure 1:**
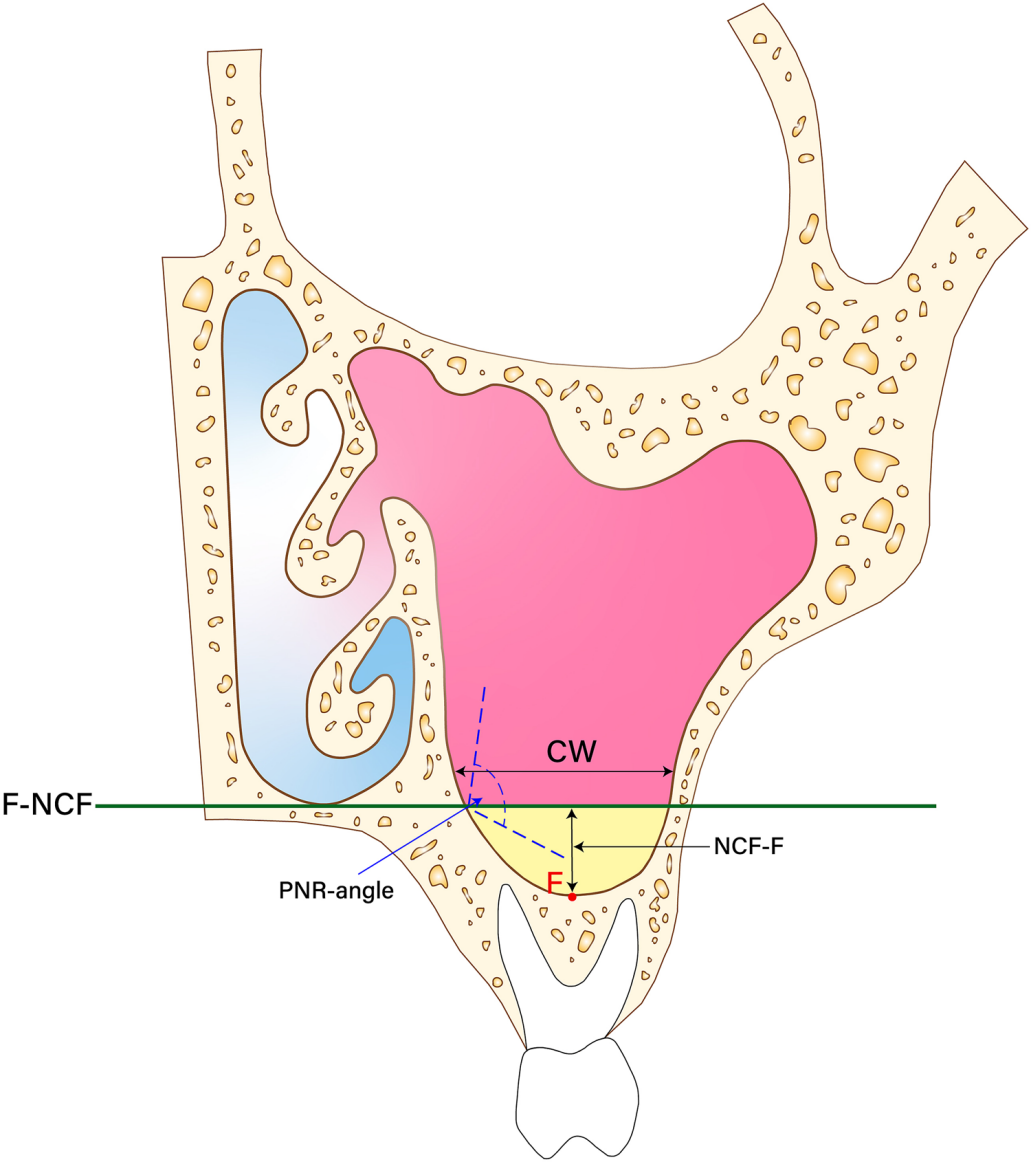
Coronal view. maxillary sinus Floor (F), nasal cavity floor (NCF), height of the inferior part of the maxillary sinus from the F to the NCF (NCF-F), width of the inferior part of the maxillary sinus in the coronal view (CW), Angle of the palatal bone and nasal bone wall (PNR angle).

**Figure 2:**
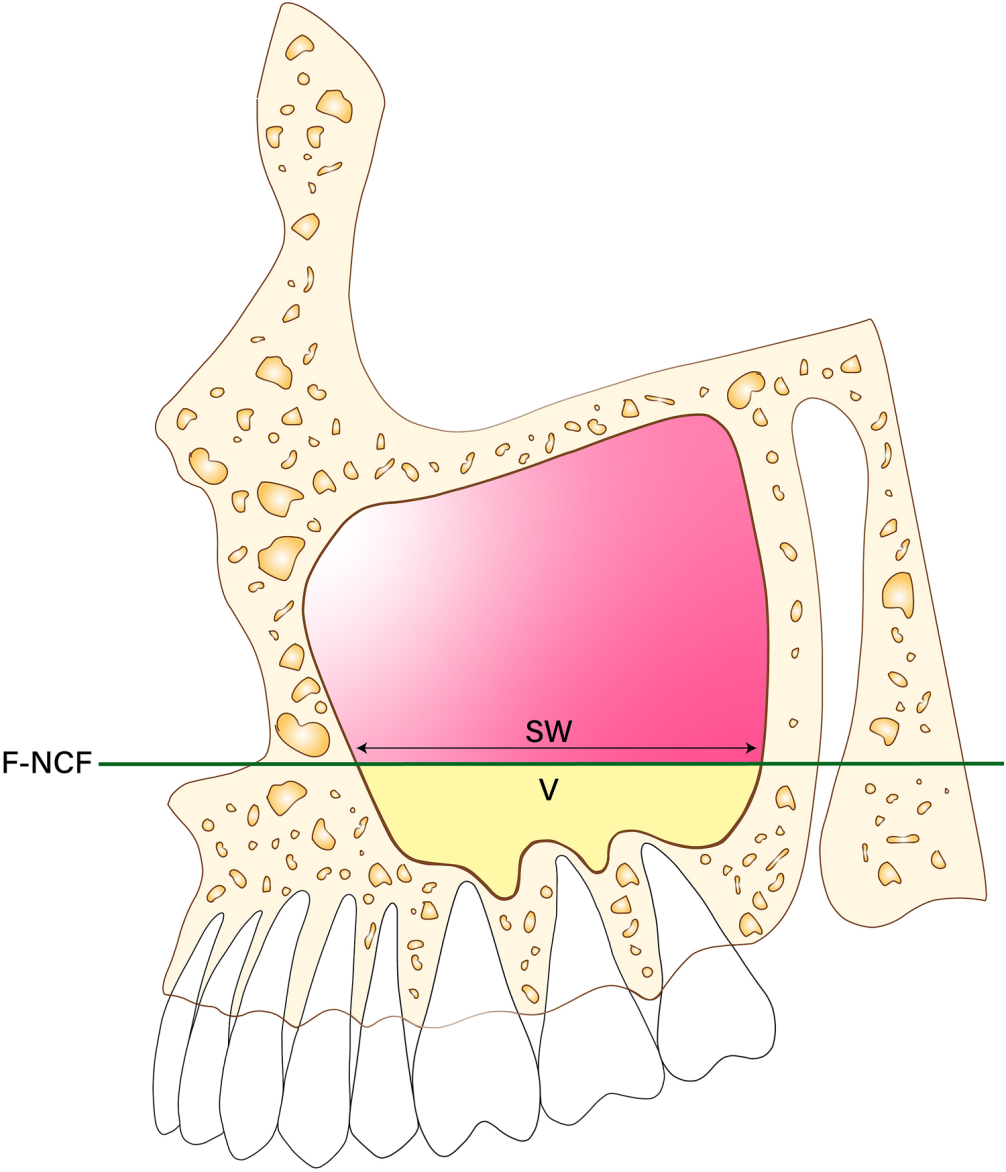
Sagittal view. nasal cavity floor (NCF), width of the inferior part of the maxillary sinus in the sagittal view (SW), volume between the inferior part of the maxillary sinus from the F to the NCF (V).

**Figure 3:**
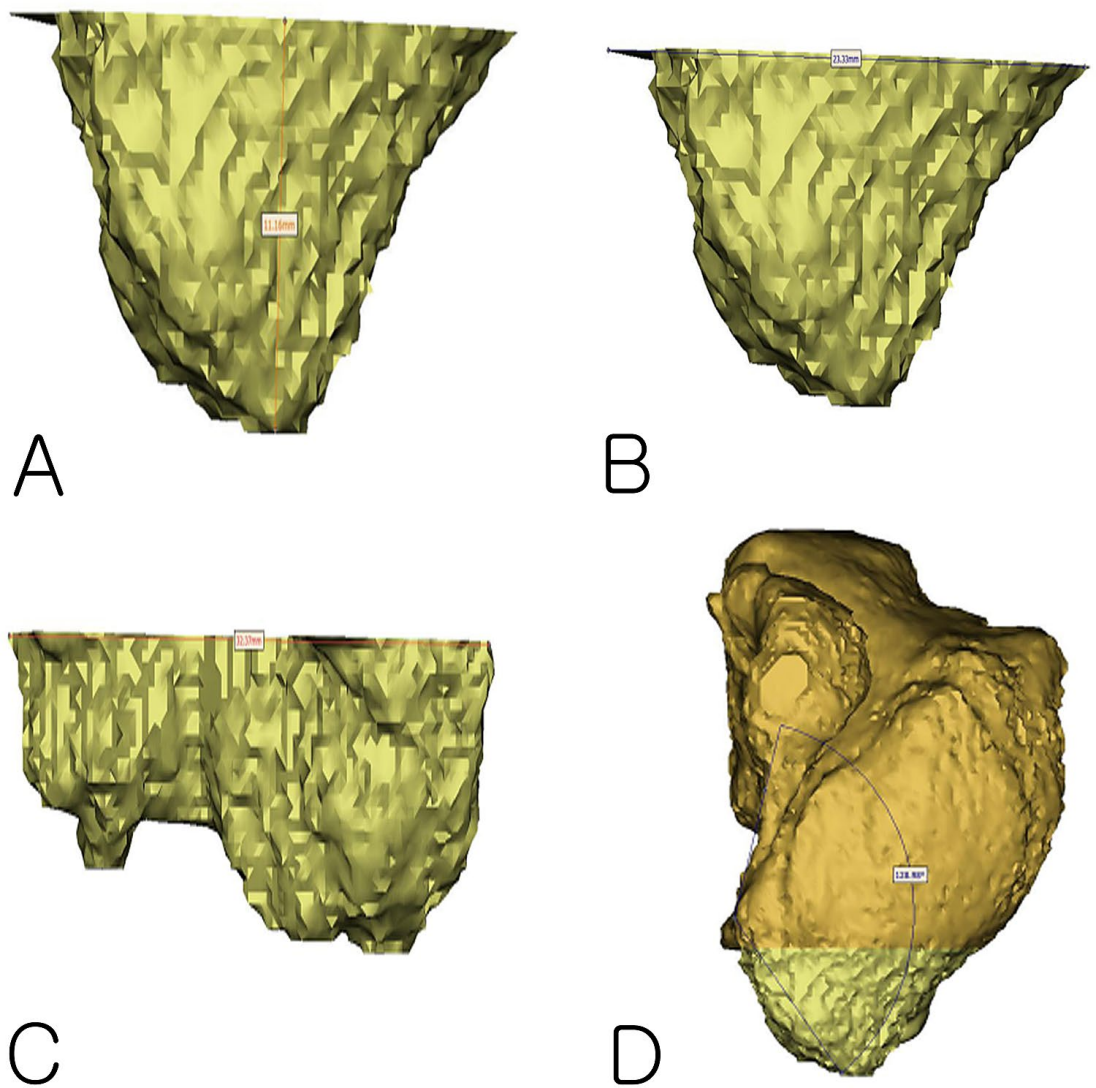
3D modeling inferior part of the maxillary sinus, (**A**) height of the inferior part of the maxillary sinus from the F to the NCF, (**B**) width of the inferior part of the maxillary sinus in the coronal view, (**C**) width of the inferior part of the maxillary sinus in the sagittal view, (**D**) Angle of the palatal bone and nasal bone wall.

To analyze the inferior part of the maxillary sinus from both the X- and Z-axes, it was divided based on the nasal cavity floor (NCF) line according to Kawakami et al.^12^. The coronal view (C-axis) and sagittal view (S-axis) of the inferior part of the maxillary sinus were analyzed as listed in Table 1 (Fig. 1), (Fig. 2), (Fig. 3).

**Table 1:** Parameters of the inferior part of the maxillary sinus.

Parameter	Definition
NCF-F	Height of the inferior part of the maxillary sinus, from the floor to the NCF
CW	Width of the inferior part of the maxillary sinus from the coronal view
SW	Width of the inferior part of the maxillary sinus from the sagittal view
V	Volume between the maxillary sinus floor and the NCF
PNR angle	Angle of the palatal bone and nasal bone wall

NCF, nasal cavity floor; PNR, palatal-nasal recess.

**Figure 4:**
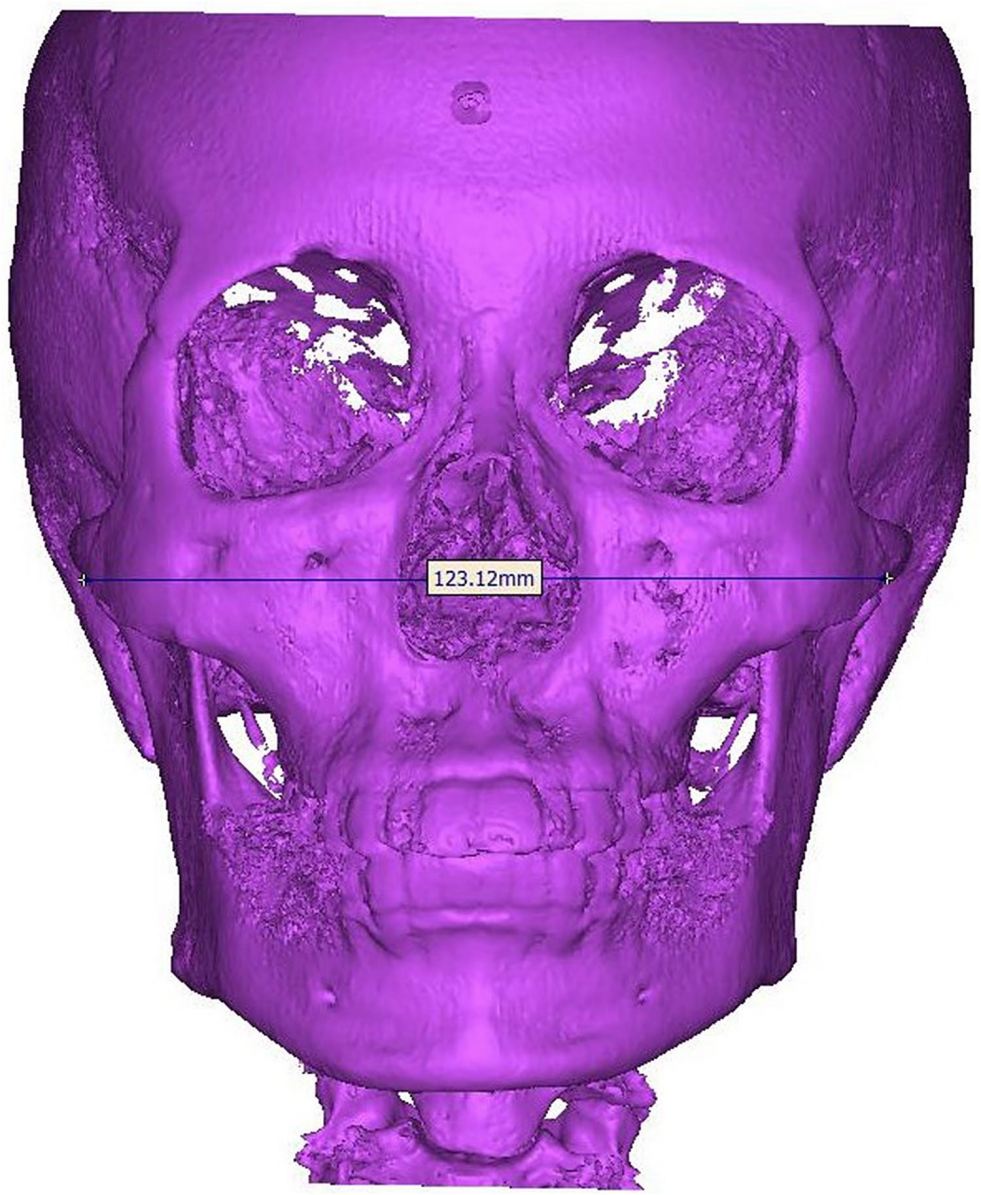
Bizygomatic breadths measurement.

To assess the inferior part of the maxillary sinus in relation to the facial width, the bizygomatic breadth was measured as the maximum linear distance between the zygions on CBCT. On the basis of the result, the patients were divided into two groups^13^: bizygomatic breadths of ≥ 127 and < 127 mm. Among the males, 14 patients were included in the ≥ 127-mm group and 16 in the < 127-mm group, while among the females, 17 patients were included in the ≥ 127-mm group and 13 in the < 127-mm group (Table 2), (Fig. 4). In order to ensure the accuracy of the measurements in the present study, two researchers (Lee, Park) recorded the measurements. After comparing their averages, statistical analysis was conducted.

**Table 2:** Bizygomatic breadths.

Measurements	Males	Females
≥ 127	14	17
< 127	16	13

#### Statistics

The measured parameters were analyzed using the Statistical Package for the Social Sciences (SPSS version 23.0, IBM, New York). Since the number of participants was small, significance was validated using the normality test followed by the Mann-Whitey U test. Post-hoc analysis was conducted at a 95% confidence interval, and differences in the mean values between males and females were validated. In addition, linear regression analysis was performed to find out whether bizygomatic breadths according to gender had an effect on the inferior portion of the maxillary sinus floor. The threshold for statistical significance was 0.05.

### Ethics approval

The study was conducted after IRB (Dankook University Dental Hospital, approval no. DUDH IRB 2015-12-022) had been approved. This study is a retrospective analysis of radiological imaging data which were obtained from the completed check-up processes. Thus, an application for waiver of consent was requested and was approved by the institutional review committee of Dankook University Dental Hospital.

### Related guidelines and regulations

This study was conducted with the methods in accordance with relevant guidelines and regulations.

## Results

The parameters of the inferior part of the maxillary sinus were measured according to sex. The heights of the inferior part of the maxillary sinus from the floor to the NCF (NCF-F values) were 15.06 mm and 13.30 mm on the left and right sides respectively in males, and 10.59 mm and 11.19 mm in females. NCF-F was significantly larger in males on both sides. The widths of the inferior part of the maxillary sinus from the axial view (CW values) were 24.49 mm and 24.26 mm on the left and right sides respectively in males, and 20.65 mm and 20.31 mm in females. CW was significantly larger in males on both sides. The widths of the posterior part of maxillary sinus from the sagittal view (SW values) were 33.64 mm and 31.34 mm on the left and right sides respectively in males, and 26.19 mm and 26.10 mm in females. SW was also significantly larger in males on both sides (p > 0.05) (Table 3).

**Table 3:** Differences in NCF-F, CW, and SW according to sex.

Parameter	Males (n = 30)	Females (n = 30)	p-value
**NCF-F (mm)**			
Left	15.06(4.54)	10.59(3.60)	< 0.001***
Right	13.30(3.39)	11.19(5.64)	< 0.05*
**CW (mm)**			
Left	24.49(5.43)	20.65(5.32)	< 0.05*
Right	24.26(7.54)	20.31(5.64)	< 0.05*
**SW (mm)**			
Left	33.64(6.15)	26.19(8.29)	< 0.001***
Right	31.34(7.04)	26.10(7.09)	< 0.05*

Data are mean (standard-deviation values).

P-value were obtained by Mann-Whitey U test.

* p < 0.05, *** p < 0.001.

The volumes between the maxillary sinus floor and the NCF (V values) were 27.05 mm^3^ and 22.55 mm^3^ on the left and right sides respectively in males, and 17.84 mm^3^ and 15.04 mm^3^ in females. V was significantly larger in males on both sides. The PNR angle was 119.42° and 119.97° on the left and right sides respectively in males, and 121.72° and 120.87° in females. The PNR angle appeared to be larger in females on both sides, but the difference was not statistically significant (Table 4).

**Table 4:** Differences in V and PNR angle according to sex.

Parameter	Males (n = 30)	Females (n = 30)	p-value
**V (mm**^**3**^**)**			
Left	27.05(15.79)	17.84(18.54)	< 0.05*
Right	22.55(13.37)	15.04(11.72)	< 0.05*
**PNR angle (°)**			
Left	119.42(18.08)	121.72(21.42)	0.690
Right	119.97(15.97)	120.87(16.20)	0.767

Data are mean (standard-deviation values).

P-value were obtained by Mann-Whitey U test.

* p < 0.05.

With regard to the facial width, the inferior part of the maxillary sinus was compared between the ≥ 127- and < 127-mm groups in males, between the ≥ 127- and < 127-mm groups in females, and between males with < 127 mm and females with ≥ 127 mm. Regarding the comparison between the ≥ 127- and < 127-mm groups in both males and females, the inferior part of the maxillary sinus had larger values for the ≥ 127-mm group than for the < 127-mm group in general. However, on the right side, males with breadths of ≥ 127 mm and < 127 mm had the following values: NCF-F 12.9 and 13.7, CW 23.5 and 25.0, SW 30.9 and 31.7, V 22.0 and 23.1, and PNR angle 123.0 and 123.0, respectively, indicating that the < 127-mm group had larger values than the ≥ 127-mm group. Additionally, females with ≥ 127-mm and < 127-mm breadths had V of 12.5 and 18.4, respectively, with a larger value for the < 127-mm group. Regarding the comparison between males with < 127-mm bizygomatic breadth and females with ≥ 127-mm bizygomatic breadth, males with < 127-mm breadth had larger NCF-F, CW, SW, and V, whereas the PNR angle was larger in females with ≥ 127-mm breadth (Table 5).

**Table 5:** Differences in Inferior part of the maxillary sinus according to bizygomatic breadth.

Measurements	Males		Females	
	≥ 127 (n = 14)	< 127 (n = 16)	≥ 127 (n = 17)	< 127 (n = 13)
**NCF-F (mm)**				
Left	15.3(4.0)	14.9(5.1)	10.6(3.0)	10.6(4.4)
Right	12.9(3.4)	13.7(3.4)	11.6(5.6)	10.7(5.9)
**CW (mm)**				
Left	25.9(6.3)	23.3(4.4)	21.0(4.8)	20.2(6.1)
Right	23.5(6.0)	25.0(8.8)	21.3(4.4)	19.0(6.9)
**SW (mm)**				
Left	33.9(6.4)	33.4(6.1)	27.3(7.4)	24.7(9.4)
Right	30.9(7.0)	31.7(7.3)	26.9(6.0)	25.0(8.4)
**V (mm**^**3**^**)**				
Left	28.3(14.7)	26.0(17.1)	19.5(20.8)	15.7(15.6)
Right	22.0(13.4)	23.1(13.8)	12.5(9.5)	18.4(13.8)
**PNR angle (°)**				
Left	115.4(19.6)	123.0(16.5)	125.1(23.5)	117.3(18.2)
Right	123.0(13.2)	117.3(18.1)	122.3(19.1)	121.1(13.6)

Data are mean (standard-deviation values).

P-value were obtained by Mann-Whitey U test.

Simple linear regression was conducted to understand if the distance between the bizygomatic breadths has an influence on the inferior portion of the maxillary sinus floor. The analysis results demonstrated that the regression model is suitable on the right side at F = 118.252 (p < 0.001), and a 93% explanatory power was observed with R2 = 0.93. NCF-F showed β = (-0.041), CW β = (0.018), SW β = (0.033), V β = (-0.041), and PNR angle β = (-0.173). It was found that the distance between the bizygomatic breadths was not statistically significant (p > 0.05). However, with regard to sex, β = (-0.173) was observed, and the distance between the bizygomatic breadths was statistically significant (p < 0.01). In addition, the β ( +) values of CW, SW, and sex all increased as the distance between the bizygomatic breadths increased. On the left side, the regression model was not suitable with F = 0.466 (p > 0.5). A 5% explanatory power was observed with R2 = 0.050. NCF-F showed β = (-0.086), CW β = (-0.300), SW β = (0.072), V β = (-0.028), PNR angle β = (-0.114), and gender β = (-0.128). The distance between the bizygomatic breadths was not statistically significant (p > 0.05). In addition, NCF-F and SW showed β ( +), and it increased as the distance between the bizygomatic breadths increased (Table 6).

**Table 6:** Impact in Inferior part of the maxillary sinus according to bizygomatic breadth.

Measurements	B	SE	*β*	t (p)	*F (p)*	*R*^*2*^
**Right**						
Constant	− 51.283	40.125		− 1.278	118.252	0.93
NCF-F (mm)	− 0.198	0.433	− 0.041	− 0.458		
CW (mm)	0.068	0.199	0.018	0.342		
SW (mm)	0.160	0.401	0.033	0.400		
V (mm^3^)	− 0.118	0.217	− 0.041	− 0.544		
PNR angle (°)	− 0.126	0.142	− 0.173	− 0.884		
Sex	66.517	21.045	0.824**	3.161**		
**Left**						
Constant	2.397	0.750		3.194	0.466	0.050
NCF-F (mm)	0.009	0.023	0.086	0.411		
CW (mm)	− 0.027	0.023	− 0.300	− 1.142		
SW (mm)	0.004	0.018	0.072	0.244		
V (mm^3^)	− 0.001	0.005	− 0.028	− 0.163		
PNR angle (°)	0.004	0.004	− 0.114	− 0.733		
Sex	0.156	0.156	− 0.128	− 0.823		

**p < 0.01.

P-value were obtained by Simple linear regression.

## Discussion

The maxillary sinus has the anatomical shape of a pyramid consisting of four walls (anterior, posterior, superior, and inferior walls) and a floor^14^. The characteristics of the inferior wall can crucially affect dental treatment^1^. In addition, the size and shape of the maxillary sinus vary^19^, and the volume of the maxillary sinus appears to be correlated with the interzygomatic buttress distance^20^. However, malocclusion factors and state of the dentition have no influence on the size of maxillary sinus^21^. Thus, while most studies have been conducted on the shape and size of maxillary sinus according to gender^3,6–12,14–16^, studies on the inferior part of the maxillary sinus are insufficient. Furthermore, accurate studies are required since the inferior part of the maxillary sinus is a critical structure that can cause complications during implant surgery. According to the study by Khairnar et al.^22^, it was reported that there were no predicted implant failure and infection symptoms when bone graft was performed on the inferior part of the maxillary sinus based on NCF. Hence, in this study, the inferior part of the maxillary sinus was classified based on the NCF, and this study was conducted to compare differences between genders and the results from the previous studies through 3-D visualization. The main results of this study are discussed below.

In this study, men were shown to be generally larger than women in the NCF-F, CW, SW, and volume categories of the maxillary sinus (p > 0.05). This was similar to the results of the study by Yoon et al.^15^, which revealed that the total volume of the maxillary sinus was larger in size in men (18.0 ml) than in women (11.1 ml). Consequently, we could confirm that the difference between men and women also appeared in the inferior part of maxillary sinus.

The comparison results between the present study and previous studies are as follows. The length of the F- NCF line was measured to determine the height of the inferior part of the maxillary sinus, and the results showed that it was longer in males on both the left and right sides, which is consistent with the results of Cavalcanti et al.^6^. Kim et al.^7^ reported that the maxillary sinus and inferior wall are 3-D structures in humans and therefore cannot be characterized properly using 2-D imaging.

The measured CW, SW, and V were all larger in males on both the left and right sides in this study, whereas Kawakami et al.^3^ reported that these three parameters were larger in females. This discrepancy is most likely due to differences between Koreans and westerners. Yoon et al.^15^ found that the maxillary sinus was larger in Koreans than in westerners. Therefore, the different results obtained in the present study compared to the study of Kawakami et al. could be attributed to the size difference in the inferior part of the maxillary sinus between Asians and westerners.

In our study, the PNR angle was larger in females than males on both sides, which is consistent with the findings of Kawakami et al.^3^. The PNR angle is an indicator of the detachment of the Schneiderian membrane during maxillary sinus floor augmentation. Chan et al.^16^ reported that a PNR angle of < 90° can lead to perforation of the Schneiderian membrane. However, since the PNR angle was measured in 2-D in previous studies, we suggest that it would be difficult to measure the maximum angle. In our study, the PNR angle was measured accurately based on the maximum angle found in 3-D visualizations.

In this study, the inferior part of the maxillary sinus was compared in relation to the facial width based on the measured bizygomatic breadth distance. The result of the comparison between the males with ≥ 127- and < 127-mm bizygomatic breadths showed that for the inferior part of the maxillary sinus on the right side, the < 127-mm group had larger NCF-F, CW, SW, and V than the ≥ 127-mm group, whereas the PNR angle was larger in the ≥ 127-mm group than in the < 127-mm group. Whether this result was in agreement with those of other studies could not be verified owing to the lack of previous studies. The result of the comparison between females with ≥ 127- and < 127-mm bizygomatic breadths showed that for the inferior part of the maxillary sinus on the right side, the ≥ 127-mm group had larger values of NCF-F, CW, SW, and PNR angle, whereas the < 127-mm group had a larger V value. The comparison between males with < 127-mm bizygomatic breadth and females with ≥ 127-mm bizygomatic breadth for the inferior part of the maxillary sinus on the right and left sides showed larger PNR angles in females with ≥ 127-mm bizygomatic breadths than in males with < 127-mm bizygomatic breadths, although the difference was not significant. Thus, the inferior part of the maxillary sinus varies in size according to facial width.

The results of the present study obtained from 3-D visualizations using CBCT data showed that the inferior part of the maxillary sinus was mostly larger in males than in females. In addition, it was found to be accurate in 3-D visualization data because the results of this study were generally similar, but statistically significant compared to previous studies. Therefore, care must be taken regarding the sex of the patient when maxillary sinus floor augmentation is performed in the future. In particular, because the PNR angle is greater in female patients than in male patients, precaution must be taken during the detachment of the Schneiderian membrane in male patients to prevent perforation. In addition, the inferior portion of the maxillary sinus showed different sizes according to the comparisons performed regarding the distance between bizygomatic breadths. Thus, when maxillary sinus floor augmentation is performed, the distance between the bizygomatic breadths should be taken into consideration. However, because the regression analysis demonstrated that only the distance between the bizygomatic breadths on the right side had a significant effect with regard to sex, a larger sample size in future studies may lead to significant results. The present study could be used as a reference for preventing complications while performing maxillary sinus floor augmentation as well as surgical procedures related to the inferior part of the maxillary sinus.

In this study, the data was not statistically significant because the number of male and female participants was not the same. In the future, it is expected that similar studies should be conducted with the same number of male and female participants so that further studies utilizing 3-D visualization will be able to yield useful fundamental data and guidelines for future research.
